# Supplementary material for: Comprehensive Characterization and In Vitro Functionality Study of Small Extracellular Vesicles Isolated by Different Purification Methods from Mesenchymal Stem Cell Cultures
Source: Int J Mol Sci. 2025 Oct 30;26(21):10602. doi: 10.3390/ijms262110602 (PMC12608249; doi:10.3390/ijms262110602)
Supplement: Supplementary file 1 [file ijms-26-10602-s001.zip › ijms-3935946-supplementary.pdf]

Proteomic analysis.

Using the one-peptide threshold, a total of 194 proteins were identified in SEC-sEVs and 161 proteins in DG-UC-sEVs. Jaccard coefficient, indicating similarity index between samples, was 46.69% (Figure S1A). Narrowing the analysis to EV-related proteins, based on ExoCarta database, 107 proteins were identified in SEC-sEVs and 99 proteins in DG-UC-sEVs. Jaccard coefficient was 51.47% (Figure S1B).

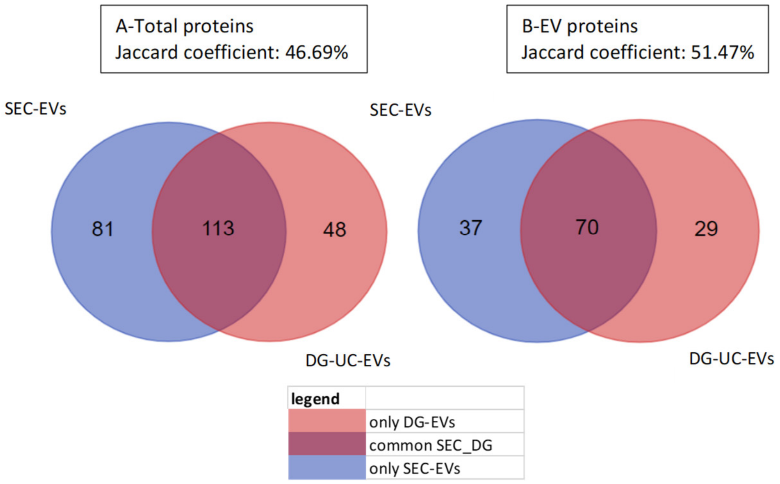

Figure S1. Venn charts of total proteins (A) and only EV-related proteins (B) detected in SEC-sEVs, in DG-UC-sEVs and in common between the two groups. Samples have been run in duplicate and analysis has been done considering 1 peptide.

Considering also proteins identified with 1 peptide, Biological Process and Biological Pathways GO analysis have been done for the proteins commonly detected in SEC-sEVs and DG-UC-sEVs (113 proteins) (Figures S2-S3).

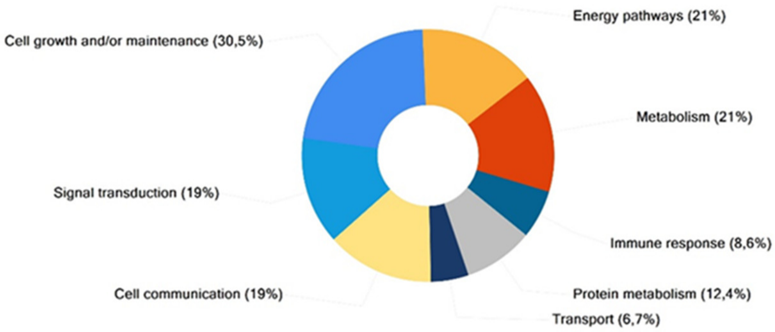

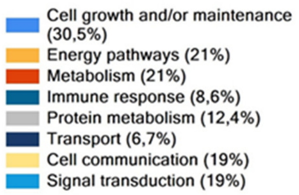

Figure S2. Biological process analysis of proteins found in common between SEC-sEVs and DG-UC-sEVs. Samples have been run in duplicate.

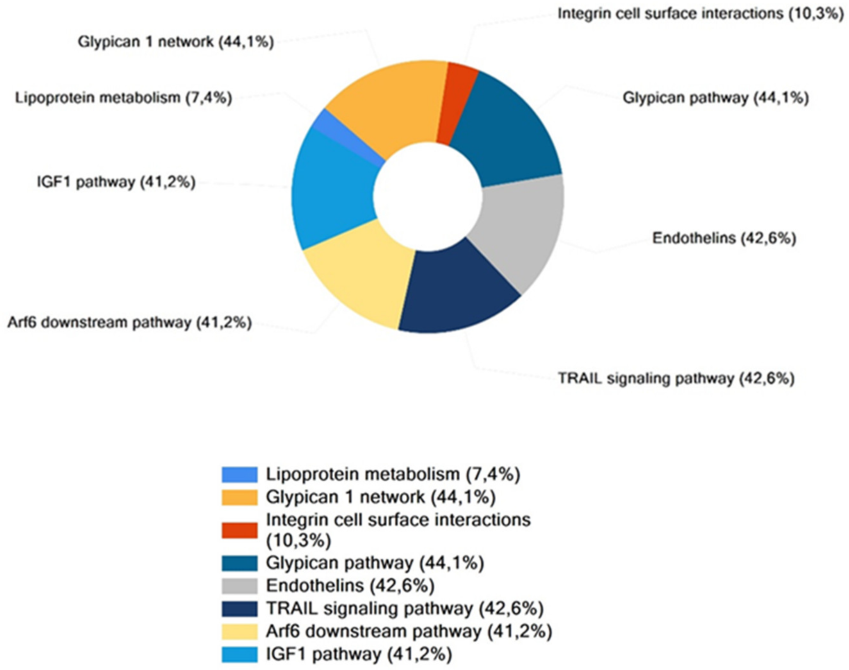

Figure S3. Biological pathways analysis of proteins found in common between SEC-sEVs and DG-UC-sEVs. Samples have been run in duplicate.

Table S1 reports the 31 proteins that are up-regulated in SEC-sEVs compared to DG-UC-sEVs, and up-regulated in DG-UC-sEVs compared to SEC-sEVs. SEC-sEVs are enriched in proteins involved in cell adhesion (BGH3, EMILIN1), innate immunity (C1RL) and inflammatory response (ATRN). DG-UC-sEVs are enriched in proteins involved in cell growth (HTRA1), cell adhesion (EDIL3), cell differentiation (AMPN), angiogenesis (AMPN; EDIL3). The two sEV samples contain few proteins which can exert functional effects, once used to treat endothelial cells. In addition, DG-UC-sEVs are enriched in proteins specifically involved in angiogenesis.

Table S1. Proteins up-regulated in SEC-sEVs compared to DG-UC-sEVs, and DG-UC-sEVs compared to SEC-sEVs. Samples have been run in duplicate.

ID	Description	Species	Genes	Significance
EDIL3_HUMAN	EGF like repeats and discoidin domains 3	Homo sapiens	EDIL3	up_DG-UC-sEVs
ATRN_HUMAN	Attractin	Homo sapiens	ATRN	up_SEC-sEVs
APOM_HUMAN	Apolipoprotein M	Homo sapiens	APOM	up_SEC-sEVs
CERU_HUMAN	Ceruloplasmin	Homo sapiens	CP	up_SEC-sEVs
HPTR_HUMAN	Haptoglobin-related protein	Homo sapiens	HPR	up_SEC-sEVs
ANGT_HUMAN	Angiotensinogen	Homo sapiens	AGT	up_SEC-sEVs
APOH_HUMAN	Beta-2-glycoprotein I	Homo sapiens	APOH	up_SEC-sEVs
A2GL_HUMAN	Leucine-rich alpha-2-glycoprotein	Homo sapiens	LRG1	up_SEC-sEVs
ALBU_HUMAN	Albumin	Homo sapiens	ALB	up_DG-UC-sEVs
TRFE_HUMAN	Serotransferrin	Homo sapiens	TF	up_DG-UC-sEVs
K2C1_HUMAN	Keratin type II cytoskeletal 1	Homo sapiens	KRT1	up_DG-UC-sEVs
PAI1_HUMAN	Plasminogen activator inhibitor 1	Homo sapiens	SERPINE1	up_DG-UC-sEVs
THBG_HUMAN	Thyroxine-bindingglobulin	Homo sapiens	SERPINA7	up_SEC-sEVs
HPLN1_HUMAN	Hyaluronan and proteoglycan link protein 1	Homo sapiens	HAPLN1	up_SEC-sEVs
K1C10_HUMAN	Keratin type I cytoskeletal 10	Homo sapiens	KRT10	up_DG-UC-sEVs
K2C5_HUMAN	Keratin, type II cytoskeletal 5	Homo sapiens	KRT5	up_DG-UC-sEVs
AMPN_HUMAN	Aminopeptidase N	Homo sapiens	ANPEP	up_DG-UC-sEVs
CBPN_HUMAN	Carboxypeptidase N catalytic chain	Homo sapiens	CPN1	up_SEC-sEVs
CO5A1_HUMAN	Collagen alpha-1(V) chain	Homo sapiens	COL5A1	up_SEC-sEVs
PGS1_HUMAN	Biglycan	Homo sapiens	BGN	up_SEC-sEVs
K1C9_HUMAN	Keratin, type I cytoskeletal 9	Homo sapiens	KRT9	up_DG-UC-sEVs
K22E_HUMAN	Keratin, type II cytoskeletal 2 epidermal	Homo sapiens	KRT2	up_DG-UC-sEVs
BTD_HUMAN	Biotinidase	Homo sapiens	BTD	up_SEC-sEVs
LUM_HUMAN	Lumican	Homo sapiens	LUM	up_SEC-sEVs
SPP24_HUMAN	Secreted phosphoprotein 24	Homo sapiens	SPP2	up_SEC-sEVs
BGH3_HUMAN	Transforming growth factor-beta-induced protein ig-h3	Homo sapiens	TGFBI	up_SEC-sEVs
TARSH_HUMAN	Target of Nesh-SH3	Homo sapiens	ABI3BP	up_SEC-sEVs
PDC6I_HUMAN	Programmed cell death 6-interacting protein	Homo sapiens	PDCD6IP	up_DG-UC-sEVs
HTRA1_HUMAN	Serine protease HTRA1	Homo sapiens	HTRA1	up_DG-UC-sEVs
C1RL_HUMAN	Complement C1r subcomponent-like protein	Homo sapiens	C1RL	up_SEC-sEVs
EMIL1_HUMAN	Elastin microfibril interface-located protein 1	Homo sapiens	EMILIN1	up_SEC-sEVs

miRNA GO analysis.

Analysis for enrichment pathways of the 66 deregulated miRNAs has been done with the miRNA Enrichment Analysis and Annotation Tool (miEAA) [54]. In particular, GO Cellular component (miRPathDB) analysis was carried out and identified 8 statistically significant enriched subcategories (Figure S4).

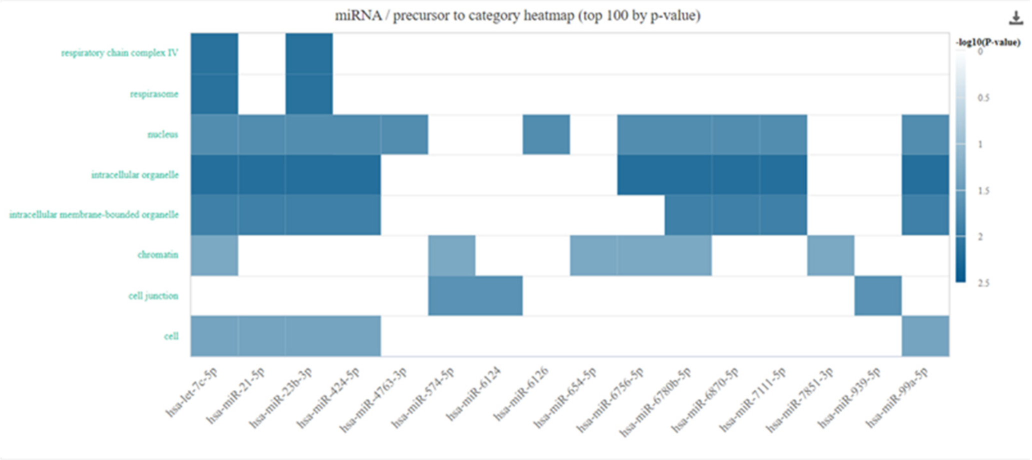

Figure S4. Heatmap showing GO CC analysis of the subset of deregulated probes found in MSC-sEVs.

Analysis with the tool Localization (RNALocate) found only two statistically significant subcategories (Cytoplasm; Nucleus). Interestingly, even if not statistically significant, more than half of total deregulated probes (37) are annotated for the following categories: Microvesicle; Exosome; Extracellular vesicle; Circulating, confirming the exosomal identity of samples (hsa-let-7c-5p; hsa-miR-1236-5p; hsa-miR-1273h-5p; hsa-miR-1275; hsa-miR-1290; hsa-miR-149-3p; hsa-miR-197-5p; hsa-miR-198; hsa-miR-199a-3p; hsa-miR-21-5p; hsa-miR-23b-3p; hsa-miR-3162-5p; hsa-miR-3197; hsa-miR-3652; hsa-miR-3940-5p; hsa-miR-424-5p; hsa-miR-4253; hsa-miR-4463; hsa-miR-4507; hsa-miR-451a; hsa-miR-4758-5p; hsa-miR-5010-5p; hsa-miR-5585-3p; hsa-miR-564; hsa-miR-574-5p; hsa-miR-6124; hsa-miR-6126; hsa-miR-6131; hsa-miR-654-5p; hsa-miR-6746-5p; hsa-miR-6821-5p; hsa-miR-6870-5p; hsa-miR-7108-5p; hsa-miR-7851-3p; hsa-miR-885-3p; hsa-miR-939-5p; hsa-miR-99a-5p).

GO Biological process (miRPathDB) and GO Molecular function (miRPathDB) analyses resulted in no enriched pathways of interest related to the functional effects of EVs (Figures S5-S6).

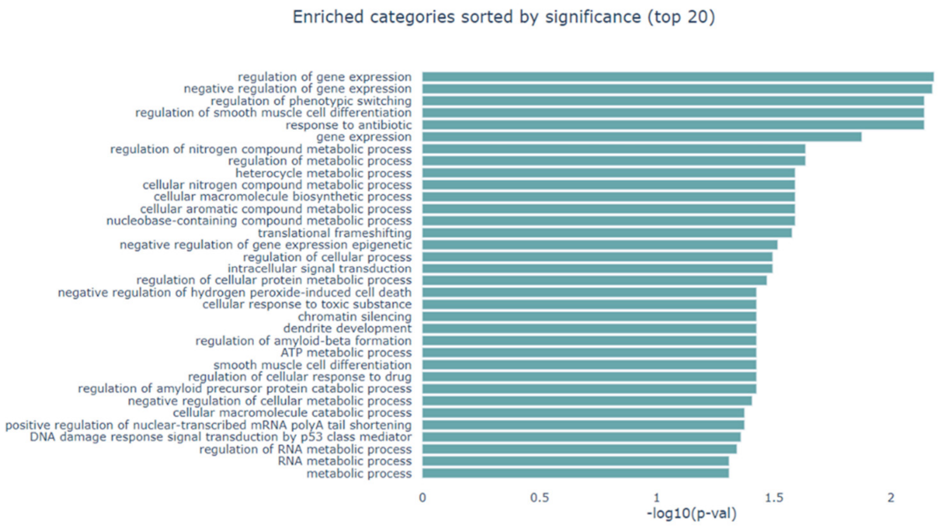

Figure S5. Heatmap showing GO Biological process analysis of the subset of deregulated probes found in MSC-sEVs.

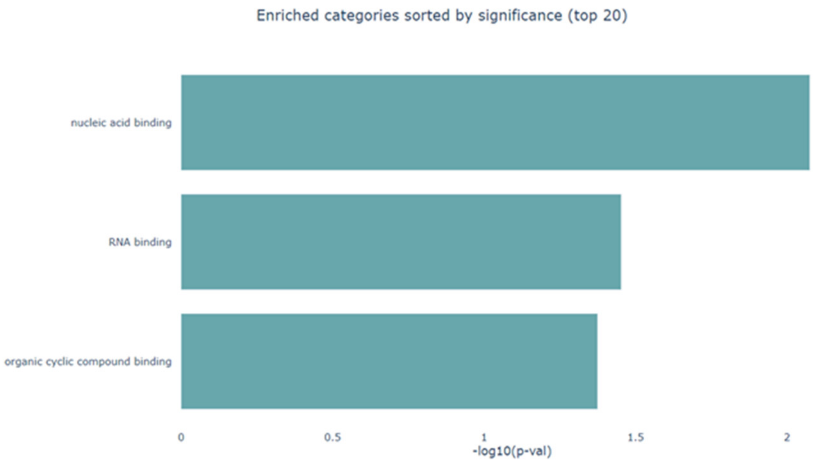

Figure S6. Heatmap showing GO Molecular function analysis of the subset of deregulated probes found in MSC-sEVs.
